# Local Wellness Policy 5 Years Later: Is It Making a Difference for Students in Low-Income, Rural Colorado Elementary Schools?

**DOI:** 10.5888/pcd10.130002

**Published:** 2013-11-07

**Authors:** Elaine S. Belansky, Nick Cutforth, Lynn Gilbert, Jill Litt, Hannah Reed, Sharon Scarbro, Julie A. Marshall

**Affiliations:** Author Affiliations: Nick Cutforth, University of Denver Morgridge College of Education; Lynn Gilbert, Jill Litt, Hannah Reed, Sharon Scarbro, Julie A. Marshall, University of Colorado Denver, Denver, Colorado

## Abstract

**Introduction:**

The federally mandated Local Wellness Policy (LWP) was intended to promote student health in schools. This study assesses the 5-year effects of the LWP on the health practices of rural elementary schools in Colorado.

**Methods:**

One year before and 5 years after the LWP mandate, a survey was administered to a random sample of principals, physical education (PE) teachers, and food-service managers in 45 rural, low-income elementary schools in Colorado. Response rates were 71% in 2005 and 89% in 2011.

**Results:**

Minutes for PE and recess did not increase, nor did offerings of fresh fruits and vegetables. More schools adopted policies prohibiting teachers from taking recess away as punishment (9.7% in 2005 vs 38.5% in 2011, *P* = .02) or for making up missed instructional time, class work, or tests in other subjects (3.2% in 2005 vs 28.2% in 2011, *P* = .03). More schools scheduled recess before lunch (22.6% in 2005 vs 46.2% in 2011, *P* = .04) and developed policies for vending machines (42.9% in 2005 vs 85.7% in 2011, *P* = .01) and parties (21.4% in 2005 vs 57.9% in 2011, *P* = .004).

**Conclusion:**

Changes in school practices are modest, and arguably the important school practices such as increased PE and recess time and increased offerings of fruits and vegetables in the lunch line have not changed in the 5 years since the mandate went into effect. Further investigation is needed to identify the knowledge, skills, and attitudes as well as financial and physical resources required for school administrators to make changes in school practices.

## Introduction

Approximately 3 of 10 children in the United States are overweight or obese and therefore at greater risk for obesity in adulthood (1) and for chronic diseases such as heart disease, diabetes, and cancer (2). Although national childhood obesity rates are stable (3), rates are increasing in Colorado (9.9% of children aged 10 to 17 years in 2003 and 14.2% in 2007) (4), and rural children have higher overweight rates than urban children (28.8% vs 20.5%) (5). Public schools are important settings for promoting healthy behaviors and reversing obesity trends (6) and this is particularly true in rural settings because of the higher prevalence of poverty (7), lower population density (8), fewer opportunities for physical activity (9), and greater distances to reach organized activity opportunities (10). Recess and physical education (PE) are sometimes the sole opportunities for rural children to engage in physical activity (11).

In response to the childhood obesity epidemic, the US government issued a mandate under the Child Nutrition and Women, Infants, and Children Reauthorization Act of 2004 requiring school districts participating in the National School Lunch Program to create a Local Wellness Policy (LWP) by June 2006 (12). The intent of the LWP was to address childhood obesity by increasing opportunities for healthy eating and physical activity. In 2010, the LWP mandate was reauthorized under the Healthy, Hunger-Free Kids Act (13) and included several new requirements: districts were told to have language in their wellness policy about goals for nutrition promotion; districts were instructed to assemble a broader team of community members to assist in developing, implementing and evaluating the wellness policy; and districts were instructed to conduct ongoing assessments of policy implementation and evaluation. The 2010 Act required the US Department of Agriculture provide technical assistance to state and local education agencies to assist school districts in complying with the mandate.

The initial effect of the LWP mandate on school environment and policy features that had been shown in previous studies to be associated with increased student physical activity and healthy eating was modest; small gains were achieved related to healthy eating (14) and no gains related to physical activity were reported (15) after its first year of implementation in rural, low-income Colorado elementary schools. LWPs were weakly worded and rarely addressed certain key issues such as calorie content of school foods. The same pattern was found in other state and national studies (16–23). This pattern could be due in part to a lag time between adopting and implementing the LWP (22).

To date, no findings have been published about the LWP’s effect on school environments and policy features since its reauthorization in 2010. The goal of this study was to assess the extent to which student opportunities for physical activity and healthy eating changed in low-income, rural schools 5 years after the LWP mandate first went into effect and 1 year after the LWP mandate was reauthorized.

## Methods

### Study sample

A random sample of 45 elementary schools was selected from 72 eligible schools in fall 2005. Eligibility criteria included being located in rural Colorado (where “rural” is defined as districts with fewer than 6,000 students and located in an area designated by the Colorado Department of Education as being either a rural, outlying town, or outlying city setting) and having at least 40% of students that qualify for free or reduced-cost lunch. The sample included 40 different school districts (1 district had 4 schools; 2 districts each had 2 schools). Colorado has 178 school districts and 1,780 schools.

### School environment and policy survey

To track school-level environment and policy features related to physical activity and healthy eating, the Rocky Mountain Prevention Research Center (RMPRC) created the School Environment and Policy Survey (SEPS) in 2005. Items were selected from the following tools and then modified to detect change over time: the Centers for Disease Control and Prevention’s (CDC’s) School Health Index (24), the Michigan Healthy School Assessment Tool (25), School Health Policies and Practices Study (26), and Eat Smart Guidelines section of the Child and Adolescent Trial for Cardiovascular Health (CATCH) Foodservice Survey (27). SEPS is a 3-module questionnaire designed to assess and track changes in the physical activity and nutrition features of a school. Principals completed Module 1, “Elementary School Policies and Factors Related to Physical Activity and Food”; food-service managers completed Module 2, “Nutrition Services”; and PE teachers completed Module 3, “Physical Education and Other Physical Activity Programs.” Examples of items include number of minutes of recess per week, minutes of PE, playground features, total number of fruit and vegetable offerings at breakfast and lunch, presence of a school health team, and familiarity with their district’s LWP and related state or federal mandates. In the principal module, respondents were asked to categorize the presence and enforcement of policies about physical activity and nutrition content of items sold in schools as follows:

1. No policy exists, written or unwritten.
2. There is an unwritten policy that is always or almost always enforced.
3. Written policy exists but is never or almost never enforced.
4. Written policy exists and is sometimes enforced.
5. Written policy exists and is always or almost always enforced.

More than 90% of respondents selected option 1 or 2. There were no instances in which respondents selected options 3 or 4. Option 5 was selected only 6% to 8% of the time, depending on the specific item. Thus, for analysis purposes, response options 2 and 5 were collapsed into 1 category, “written or unwritten policy exists that is always or almost always enforced.” Study authors collected validation data on the SEPS instrument in 2007 by comparing the self-reported measures with direct observations of 10 rural and low-income elementary schools that were not part of the random sample. Initial analyses suggested minimal reporting bias (15). For example, independent observers found that time spent in PE classes was less than 1 minute shorter on average than the duration reported by PE teachers on the survey. Additional analyses are needed to complete the validation study. School personnel completed the SEPS in fall 2005, 8 months prior to the deadline for districts to have an LWP in place and 6 months after Colorado passed bill SB 05-081 (28), which encouraged school boards to adopt the federally mandated wellness policy. The survey was repeated 3 times after the LWP went into effect: fall 2006, fall 2007, and fall 2011. New analyses presented here compare 2005 and 2011 to address longer-term effect, and previously published early findings for 2007 (14,15) are included for comparison. The Colorado Multiple Institutional Review Board approved the study protocol.

The Dillman Method (29) was used in all years to optimize survey response rates: each school received a prenotice phone call 2 days before the survey was mailed. The mailing included the 3 survey modules along with a $50 gift card to Target or Walmart retail stores. Reminder phone calls were made to the principal or school secretary 3 days after the requested return date and once a month after that until the survey was returned with a maximum of 4 follow-up contacts in the form of voicemail messages left with the principal or messages left with the secretary.

Analyses were performed using SAS (version 9.2, 2008, SAS Institute Inc, Cary, North Carolina). A *P* value of <.05 was considered significant. To test for trends with a binary response variable, the Generalized Estimating Equations with a binomial distribution, logit link, and compound symmetry correlation structure was used. In the case of a continuous variable, the General Linear Mixed Model with the maximum likelihood estimation method for the covariance parameters and a variance components covariance structure was used. Both types of analyses used a random-effects model that allowed for an unbalanced design; that is, schools with data for either or both years were included to increase study power. The models for both binomial and continuous variables included a random school effect (eg, number of daily fresh-fruit lunch choices year 2005 vs year 2011 + random school effect). Models testing 2005 versus 2011 did not include 2007. The 2007 data (Table 2, Table 3) are shown to help the reader see trends over time, but the data comparing 2005 and 2007 were published elsewhere (14,15). Additional analyses, which restricted the dataset to schools with data at both time points (n = 26), were conducted to ensure that estimates of trends over time were not biased by the unbalanced design. Although statistical power was reduced, the results were similar and therefore are not presented here. Of the 26 schools for which we had survey data in 2005 and 2011, more than 50% had the same food-service and PE teacher respondents. However, 65% of principals changed from 2005 to 2011.

## Results

### School demographics

Among the 45 schools in the random sample, student enrollment ranged from 28 to 546 (mean, 204); students receiving free or reduced-price lunch rates ranged from 40% to 82% (mean 55%); and student body Hispanic ethnicity ranged from 0% to 72% (mean 27%). Survey response rates ranged from 71% to 91%. The demographics of the universe of 72 eligible schools are similar to the 45 randomly selected schools: 215 students on average per school, 55% of students qualifying for free or reduced-price lunch, and 28% of students Hispanic (Table 1).

**Table 1:** Rural Random Sample School Participation Rates and Demographic Characteristics, Colorado

Variable	Random Sample (N = 45)	Schools Completing SEPS in Fall 2005 (n = 32)	Schools Completing SEPS in Fall 2006 (n = 41)	Schools Completing SEPS in Fall 2007 (n = 38)	Schools Completing SEPS in Fall 2011 (n = 40)
Participation rate	NA	71%	91%	84%	89%
% Students receiving free/reduced-price lunch, mean (SD)	54.4 (10.5)	55.2 (9.6)	54.4 (10.6)	54.8 (11.1)	54.3 (9.9)
% Hispanic	27.0 (21.5)	24.8 (21.2)	28.7 (22.1)	29.5 (22.0)	28.3 (21.3)
Mean (SD) number of students per school	204.1 (144.7)	218.1 (157.4)	214.3 (149.1)	211.5 (144.2)	215.0 (148.4)

Abbreviation: NA, not applicable; SEPS, School Environment and Policy Survey; SD, standard deviation.

### School environment and policy trends related to physical activity

Effective practices associated with physical activity were examined 5 years after the LWP went into effect. These analyses considered policies and practices reported at the school level by principals and physical education teachers (Table 2). The amount of time for physical education and recess had not increased since the LWP went into effect. However, other gains had been made for school recess including more policies in place prohibiting classroom teachers from withholding recess as a punishment and withholding recess to make up missed instructional time, class work, or tests in other subjects. We saw a positive nonsignificant trend indicating teachers or recess monitors were setting up games and activities during recess more frequently than they were 5 years prior. We found no policy changes protecting physical education time.

**Table 2:** Trends in School-Level Physical Activity Features in Rural, Low-Income Colorado Elementary Schools

School Physical Activity Feature	Fall 2005 (Before LWP)	Fall 2007 (1 Year After LWP)	*P* value, 2005 vs 2007	Fall 2011 (5 Years After LWP)	*P* value, 2005 vs 2011^a^
**Physical education**					

Weekly physical education minutes for 5th graders, no. of schools	29	37	.10	38	.20
Mean (SD), range	102.7 (51.7) 45–240	119.9 (67.4) 50–315		113.8 (54.0) 50–300	

**Physical education school-level policies^b^**					

Prohibits classroom teachers from withholding physical education class as a punishment, no. of schools	31	38	.50	39	.70
No school policy, %	45	37		39	
Written or unwritten school policy, %	55	63		62	

Prohibits classroom teachers from withholding physical education class to make up missed instructional time, class work, or tests in other subjects, no. of schools	31	38	.40	39	.70
No school policy, %	42	50		44	
Written or unwritten school policy, %	58	50		56	

Prohibits replacement of physical education class with other activities, no. of schools	31	38	.70	39	.80
No school policy, %	55	50		51	
Written or unwritten school policy, %	45	50		49	

**Recess**					

Daily recess minutes for fifth graders	31	37	.10	39	.13
Mean (SD)	36.9 (11.1)	33.1 (11.4)		33.7 (11.7)	

Teachers or recess monitors set up games and activities during recess, no. of schools	31	38	.40	39	.08
Sometimes, %	55	55		74	
Never, %	45	45		26	

**Recess school-level policies^b^**					

Offers daily opportunities for unstructured physical activity, such as recess, lasting at least 20 minutes, no. of schools	31	38	.40	39	.70
No school policy, %	10	16		10	
Written or unwritten school policy regarding, %	90	84		90	

Prohibits classroom teachers from withholding recess as a punishment, no. of schools	31	38	.30	39	.02
No school policy, %	90	79		62	
Written or unwritten school policy, %	10	21		39	

Prohibits classroom teachers from withholding recess to make up missed instructional time, class work, or tests in other subjects, no. of schools	31	38	.30	39	.03
No school policy, %	97	90		72	
Written or unwritten school policy, %	3	11		28	

Abbreviation: LWP, Local Wellness Policy; SD, standard deviation.

a A *P* value of <.05 was considered significant. Calculated using General Estimating Equations and General Linear Mixed Model. Models testing 2005 versus 2011 did not include 2007.

b School policies are guidelines established by school principals for their schools. These policies do not refer to what is or is not included in the district-level LWP.

### School environment and policy trends related to healthy eating

Next, we examined whether effective practices associated with healthy eating had changed 5 years after the LWP went into effect. These analyses considered policies and practices reported at the school level by principals and food-service managers.

We report trends inside the lunchroom (reported by food-service managers unless otherwise noted) and outside the lunchroom (reported by principals) 1 year before (2005), 1 year after (2007), and 5 years after (2011) implementation of the LWP (Table 3). Despite an initial increase in the number of fresh fruits offered daily from 2005 to 2007, offerings declined in 2011, resulting in no statistical difference in fresh fruit offerings between 2005 and 2011. Likewise, fresh vegetable offerings did not increase since the LWP mandate went into effect. There were no changes in the number of schools offering a salad bar or getting produce from local farmers.

**Table 3:** Trends in School-Level Nutrition Features in Rural, Low Income Colorado Elementary Schools

Nutrition Features	Fall 2005 (Before LWP)	Fall 2007 (1 Year After LWP)	2005 vs 2007, *P* value^a^	Fall 2011 (5 Years After LWP)	2005 vs 2011, *P* value^a^
**Inside the lunchroom**					
No. of daily fresh fruit lunch choices, mean (SD)	30, 0.8 (0.71)	34, 1.2, (0.89)	.04	40, 1.1 (0.75)	.08
No. daily fresh vegetable lunch choices, mean (SD)	30, 1.3 (0.98)	34, 1.3 (1.03)	.90	40, 1.4 (1.08)	.90
Get produce from local farmers, no. of schools (%)	30 (30)	34 (29)	.70	40 (35)	.40
Place fruits at front of lunch line, no. of schools (%)	30 (7)	33 (15)	.20	38 (8)	.50
Place vegetables at the front of the lunch line, no. of schools (%)	30 (20)	34 (9)	.30	38 (11)	.30
Salad bar occasionally or every day, no. of schools (%)	30 (50)	35 (46)	.60	39 (44)	.20
No. of minutes allotted for fifth-grade lunch, mean (SD)^b^	31, 21.9 (6.01)	37, 23.2 (6.25)	.47	39, 22.1 (4.25)	.90
Lunchroom monitors encourage students to eat their fruits and vegetables at least 1–2 times per week, no. of schools (%)	31 (87)	35 (94)	.40	39 (97)	.10
Use offer system (vs serve system), no. of schools (%)	27 (59)	35 (57)	.90	40 (45)	.20
Offer à la carte food items, no. of schools (%)	29 (21)	34 (18)	.60	37 (19)	.20
À la carte menu offers fruits and vegetables, no. of schools (%)	6 (67)	6 (50)	.70	7 (71)	.40
À la carte menu offers candy, high-fat snacks, or high-calorie fast foods, no. of schools (%)	6 (83)	6 (50)	.10	7 (43)	.10
**Outside the lunchroom**					
Vending machines, no. of schools (%)	31 (45)	38 (53)	.40	39 (54)	.40
Of those with vending machines, policy stipulating predominantly healthy foods and beverages be offered, no. of schools (%)	14 (43)	20 (80)	.20	21 (86)	.01
Vending machines with carbonated beverages, no. of schools (%)	14 (57)	20 (45)	.30	21 (14)	.06
Vending machines with high-fat, high-calorie items, no. of schools (%)	14 (50)	20 (40)	.20	21 (19)	.40
Require healthy foods and beverages in classroom parties, no. of schools (%)	28 (21)	37 (49)	.04	38 (58)	.004
Lunch recess is before lunch, no. of schools (%)	31 (23)	38 (16)	.70	39 (46)	.04

Abbreviation: LWP, local wellness policy; SD, standard deviation.

a A *P* value of <.05 was considered significant. Calculated using General Estimating Equations and General Linear Mixed Model. Models testing 2005 versus 2011 did not include 2007.

b Principal provided this information.

Outside the lunchroom, the percentage of schools stipulating the availability of healthy foods and beverages in vending machines and at classroom parties continued to increase. In addition, the percentage of schools reversing the order in which lunch and recess are scheduled increased. Although no changes we observed in the number of schools with vending machines, of those with vending machines, the percentage of schools stipulating the availability of healthy foods and beverages increased significantly. Among schools with vending machines, there were nonsignificant trends indicating fewer schools offering carbonated beverages and high-fat and high-calorie items.

## Discussion

The LWP was intended to increase student opportunities for physical activity and healthy eating as a strategy to address the childhood obesity epidemic. These opportunities are especially needed in Colorado’s rural schools where childhood obesity rates are the highest (5). Five years after the LWP was mandated, what are arguably the most important school practices — those that can increase physical activity and healthy eating for most students on most days — have not changed: minutes allotted for PE and recess remained the same and lunchrooms are not offering more fresh fruits or vegetables. However, some modest yet noteworthy accomplishments have been made. For example, more schools have policies protecting recess, schedules in which recess takes place before lunch, and policies requiring predominantly healthy foods in vending machines and at classroom parties.

The overall comprehensiveness and strength of wellness policies have improved nationally over the past 5 years; however, both remain weak (30). Perhaps if quantifiable targets for important school practices (eg, 150 minutes of PE per week) had been part of the federal mandate or related state mandates, more progress would have been made. However, at the time the LWP was mandated, no such targets existed in Colorado. In 2005, Colorado State Bill 05-081 (28) “encouraged” school boards of education to adopt an LWP and increase opportunities for physical activity and healthy eating, but the bill did not provide quantifiable goals (28). The Colorado Association of School Boards included a specific target for physical activity in an LWP template the board created in 2005 to assist school districts in writing their LWPs: “A requirement or encouragement that periods of physical activity be at least 150 minutes per week for elementary students and at least 225 minutes per week for secondary students.” However, this language falls well below national recommendations of 150 minutes of PE per week (31) in addition to 100 minutes of recess (32) for elementary students. Five years after the LWP went into effect, Colorado passed SB 11-1069, which required elementary schools to provide at least 30 minutes per day of any type of physical activity (33). However, our data indicate that schools were already exceeding that goal with recess alone 6 years prior to passage of the bill (eg, students were getting an average of 37 minutes of recess per day in 2005 [Table 2]). Research is needed to investigate the link between specific state targets and the effect of the LWP on key school practices.

Budd et al (34) suggest that successful school policy implementation depends on a school’s organizational capacity to deal with challenges, set clear goals, and have accountability practices in place. Schools in rural, low-income areas have limited organizational capacity because of small staff size and personnel performing multiple roles and tasks (eg, the superintendent may also be the K-12 principal, who is often overloaded with new state and district policies) (15). Amis et al further suggest that a new educational environment makes it difficult for schools to implement policies related to childhood obesity prevention (35). They contend that No Child Left Behind (36) has altered the value system of schools such that the focus on providing holistic development has been replaced with a focus on academic achievement and standardized tests. They argue that for childhood obesity policies to take hold in schools, principals and stakeholders need to be educated on the link between academic achievement and physical activity. Furthermore, studies have found that the extent to which wellness policies are implemented will depend on whether school boards, superintendents, principals, and teachers are held as accountable for the quality of their schools’ physical activity and nutritional environments as they are for their students’ performance on standardized tests (15,34,35).

Schools also need technical and financial assistance for implementation. Although federal agencies such as CDC and state departments of education like Colorado’s provide schools with tools and resources to develop, implement, and evaluate their LWPs, partnerships are needed between rural schools and external groups such as a university, foundation, or nonprofit organization trained in school wellness issues to implement “big ticket” changes such as increases in PE and recess time and healthy food offerings in the lunchroom. Our university plays an active role in helping schools implement research-based practices known to increase student opportunities for physical activity and healthy eating. Our team developed a strategic planning process called AIM for evidence-based health promotion in school settings. AIM has been shown to lead to an average of 4 effective environment and policy strategies implemented per school (37). In Colorado, we are also fortunate to have the Colorado Health Foundation and LiveWell Colorado, 2 organizations that provide financial resources and technical assistance to help schools implement wellness initiatives.

Strengths of this study include its random sample of rural, low-income Colorado schools and its longitudinal design. Limitations include reliance on self-reported data from school personnel as well as limitations of the survey itself (eg, the survey did not ask about classroom activity breaks and other potential strategies schools might be using to increase physical activity and healthy eating). Because of the small sample, results may only be generalizable to rural, low-income areas of Colorado.

Modest improvements to physical activity and healthy eating opportunities have been made in Colorado in the past 5 years. These changes are in the form of policies requiring more healthy foods in vending machines and classroom parties, scheduling recess before lunch, and prohibiting removing recess as punishment or for missed work. Perhaps schools were able to make these changes because they required minimal to no implementation costs or staff resources to maintain. Even though schools succeeded in adopting these policies, we know that policies alone do not always produce change (38). Future research is needed to examine the differential effect of written, board-approved policies compared with health-promoting school practices that are not board approved but are enforced at the building level. Future research is also needed to understand the reasons schools were able to make these changes, the extent to which these policies were enforced consistently across school staff and time, and what facilitated successful enforcement. Perhaps more importantly, research is needed to better understand what knowledge, skills, and attitudes as well as financial and physical resources are needed for school administrators to make more significant changes such as increasing the quantity and quality of PE and recess and providing more fruits and vegetables in the cafeteria. University researchers, local health foundations, and state organizations that support Colorado educators should join forces in an effort to provide professional development opportunities to school administrators and personnel to increase knowledge of the link between academic achievement, physical activity and healthy eating; to build skills to increase the quality and quantity of PE, recess, and healthy food purchasing and preparation; and to set up accountability structures to ensure changes are consistently implemented. However, professional development alone will not be sufficient for schools to put the most important changes in place. Schools need additional funds to increase PE time and to purchase and prepare healthy, fresh foods.

## References

[1] Biro FM , Wien M . Childhood obesity and adult morbidities. Am J Clin Nutr 2010;91(5):1499S–505S. 10.3945/ajcn.2010.28701B 20335542 PMC2854915

[2] National Institutes of Health. Clinical guidelines on the identification, evaluation, and treatment of overweight and obesity in adults: the evidence report. Bethesda (MD): National Institutes of Health, US Department of Health and Human Services; 1998.

[3] Ogden CL , Carroll MD , Kit BK , Flegal KM . Prevalence of obesity and trends in body mass index among US children and adolescents, 1999-2010. JAMA 2012;307(5):483–90. 10.1001/jama.2012.40 22253364 PMC6362452

[4] National Survey of Children's Health. NSCH 2007. Data query from the Child and Adolescent Health Measurement Initiative, Data Resource Center for Child and Adolescent Health website. www.childhealthdata.org. Accessed December 30, 2012.

[5] Liu J , Bennett KJ , Nusrat H , Sheng X , Probst JC , Pate RR . Overweight and physical inactivity among rural children aged 10–17: a national and state portrait. South Carolina Rural Research Center, Columbia (SC); 2007.

[6] Serdula MK , Collins ME , Williamson DF , Anda RF , Pamuk E , Byers TE . Weight control practices of US adolescents and adults. Ann Intern Med 1993;119(7 Pt 2):667–71. 10.7326/0003-4819-119-7_Part_2-199310011-00008 8363194

[7] USDA Economic Research Services. January 17, 2012, State Fact Sheet: Colorado. www.ers.usda.gov/statefacts/CO.HTM . Accessed Feburary 29, 2012.

[8] Colorado Rural Health Center. Snapshot of rural health in Colorado. The State Office of Rural Health; 2011. www.coruralhealth.org/resources/documents/2011snapshot.pdf . Accessed March 15, 2012.

[9] Wilcox S , Castro C , King AC , Housemann R , Brownson RC . Determinants of leisure time physical activity in rural compared with urban older and ethnically diverse women in the United States. J Epidemiol Community Health 2000;54(9):667–72. 10.1136/jech.54.9.667 10942445 PMC1731735

[10] Moore JB , Jilcott SB , Shores KA , Evenson KR , Brownson RC , Novick LF . A qualitative examination of perceived barriers and facilitators of physical activity for urban and rural youth. Health Educ Res 2010;25(2):355–67. 10.1093/her/cyq004 20167607 PMC10170971

[11] Yousefian A , Ziller E , Swartz J , Hartley D. Active living for rural youth: addressing physical inactivity in rural communities. J Public Health Manag Pract 2009;15(3):223–31. 19363402 10.1097/PHH.0b013e3181a11822

[12] Child Nutrition and WIC Reauthorization Act of 2004, Pub L. No. 108-4981 (2004).

[13] Healthy, Hunger-Free Kids Act of 2010, Pub. L. No. 111-296 (2010).

[14] Belansky ES , Cutforth N , Delong E , Litt J , Gilbert L , Scarbro S , Early effects of the federally mandated local wellness policy on school nutrition environments appear modest in Colorado’s rural, low-income elementary schools. J Am Diet Assoc 2010;110(11):1712–7. 10.1016/j.jada.2010.08.004 21034885

[15] Belansky ES , Cutforth N , Delong E , Ross C , Scarbro S , Gilbert L , Early impact of the federally mandated local wellness policy on physical activity in rural, low income elementary schools. J Public Health Policy 2009;30( Suppl 1):S141–60. 10.1057/jphp.2008.50 19190570

[16] Action for Healthy Kids. Supporting fact sheet on preliminary analysis of local wellness policies. 2006. http://www.actionforhealthykids.org/filelib/pr/Fact%20sheet%20on %20WP%20Analysis%208%2021%202006.pdf. Accessed July 13, 2012.

[17] Metos J , Nanney MS . The strength of school wellness policies: one state’s experience. J Sch Health 2007;77(7):367–72. 10.1111/j.1746-1561.2007.00221.x 17680895 PMC3268363

[18] Probart C , McDonnell E , Weirich E , Schilling L , Fekete V . Statewide assessment of local wellness policies in Pennsylvania public school districts. J Am Diet Assoc 2008;108(9):1497–502. 18755322 10.1016/j.jada.2008.06.429

[19] Belansky ES , Chriqui JF , Schwartz MB . (2009). Local school wellness policies: how are schools implementing the congressional mandate? A Robert Wood Johnson Foundation Research Brief; June 2009. http://www.rwjf.org/childhoodobesity/product.jsp?id=44708. Accessed December 30, 2009.

[20] Chriqui JF , Schneider L , Chaloupka FJ , Ide K , Pugach O . Local wellness policies: assessing school district strategies for improving children’s health. School years 2006–07 and 2007–08. Chicago (IL): Bridging the Gap Program, Health Policy Center, Institute for Health Research and Policy, University of Illinois at Chicago; 2009.

[21] Pitt Barnes S , Robin L , O’Toole TP , Dawkins N , Kettel Khan L , Leviton LC . Results of evaluability assessments of local wellness policies in 6 US school districts. J Sch Health 2011;81(8):502–11. 10.1111/j.1746-1561.2011.00620.x 21740436

[22] Gaines AB , Lonis-Shumate SR , Gropper SS . Evaluation of Alabama public school wellness policies and state school mandate implementation. J Sch Health 2011;81(5):281–7. 10.1111/j.1746-1561.2011.00588.x 21517868

[23] Turner L , Chaloupka FJ . Slow progress in changing the school food environment: nationally representative results from public and private elementary Schools. J Acad Nutr Diet. 2012;112(9):1380–9. 10.1016/j.jand.2012.04.017 22673797

[24] Centers for Disease Control and Prevention. School Health Index: a self-assessment and planning guide; 2000. https://apps.nccd.cdc.gov/SHI/Default.aspx. Accessed March 3, 2011.

[25] Michigan Department of Community Health. Michigan healthy school assessment tool. http://www.mihealthtools.org/hsat/. Accessed August 14, 2013.

[26] Centers for Disease Control and Prevention. School Health Policies and Practices Study. http://www.cdc.gov/healthyyouth/shpps/questionnaires.htm. Accessed August 14, 2013.

[27] Osganian SK , Ebzery MK , Montgomery DH , Nicklas TA , Evans MA , Mitchell PD , Changes in the nutrient content of school lunches: Results from the CATCH Eat Smart Food Service Intervention. Prev Med 1996;25(4):400–12. 10.1006/pmed.1996.0072 8818064

[28] State of Colorado. SB 05-081. http://www.leg.state.co.us/CLICS2005A/csl.nsf/fsbillcont3/B22095692E95C60087256F4D006D5B5A?Open&file=081_enr.pdf. Accessed July 18, 2012.

[29] Dillman DA . Mail and Internet surveys: the tailored design method, 2nd edition. New York (NY): John Wiley & Sons; 2007.

[30] Chriqui JF , Resnick EA , Schneider L , Schermbeck R , Adcock T , Carrion V , School district wellness policies: evaluating progress and potential for improving children’s health five years after the federal mandate. School years 2006–07 through 2010–11.Volume 3. Chicago (IL): Bridging the Gap Program, Health Policy Center, Institute for Health Research and Policy, University of Illinois at Chicago; 2013, http://www.bridgingthegapresearch.org. Accessed August 14, 2013.

[31] National Association for Sport and Physical Education. 2012. Physical education guidelines. http://www.aahperd.org/naspe/standards/nationalGuidelines/PEguidelines.cfm. Accessed August 14, 2013.

[32] National Association for Sport and Physical Education. Recess for elementary school students. Reston (VA): National Association for Sport and Physical Education; 2006. http://www.aahperd.org/naspe/standards/upload/Recess-for-Elementary-School-Students-2006.pdf. Accessed April 25, 2013.

[33] State of Colorado. Senate Bill 11-1069. http://www.leg.state.co.us/clics/clics2011a/csl.nsf/fsbillcont3/9CF56533FEFE87598725780800800FBF?open&file=1069_enr.pdf). Accessed August 14, 2013.

[34] Budd EL , Schwarz C , Yount BW , Haire-Joshu D . Factors influencing the implementation of school wellness policies in the United States, 2009. Prev Chronic Dis 2012;9:110296. 22742592 10.5888/pcd9.110296 PMC3457767

[35] Amis JM , Wright PM , Dyson B , Vardaman JM , Ferry H . Implementing childhood obesity policy in a new educational environment: the cases of Mississippi and Tennessee. Am J Public Health 2012;102(7):1406–13. 10.2105/AJPH.2011.300414 22420819 PMC3478004

[36] No Child Left Behind Act of 2001, Pub. L. 107–10, 20 U.S.C.A., Section 6301 (2001).

[37] Belansky ES , Cutforth N , Chavez R , Crane L , Waters E , Marshall JA . Adapted Intervention Mapping: A strategic planning process for increasing physical activity and healthy eating opportunities in schools via environment and policy change. J Sch Health 2013;83(3):194–205. 10.1111/josh.12015 23343320

[38] Kelder SH , Springer AS , Barroso CS , Smith CL , Sanchez E , Ranjit N , Implementation of Texas Senate Bill 19 to Increase Physical Activity in Elementary Schools. J Public Health Policy 2009;30 Suppl 1:S221–47. 10.1057/jphp.2008.64 19190576 PMC2917835

